# Draft Genome Sequence of *Sphingomonas* sp. Strain Sph1(2015), Isolated from a Fouled Membrane Filter Used to Produce Drinking Water

**DOI:** 10.1128/genomeA.00517-17

**Published:** 2017-06-15

**Authors:** Hendrik J. de Vries, Ian P. G. Marshall, Lars Schreiber, Caroline M. Plugge

**Affiliations:** a Laboratory of Microbiology, Wageningen University, Wageningen, The Netherlands; b Wetsus, European Centre of Excellence for Sustainable Water Technology, Leeuwarden, The Netherlands; c Department of Bioscience, Center for Geomicrobiology, Aarhus University, Aarhus, Denmark

## Abstract

We report here the high-quality draft genome sequence of *Sphingomonas* sp. strain Sph1(2015), isolated from a fouled reverse osmosis membrane used for the production of high-quality drinking water. The draft sequence provides insights into the *modus operandi* of this strain to form biofilms on membrane surfaces. This knowledge offers tools to develop novel antifouling strategies.

## GENOME ANNOUNCEMENT

High-pressure membrane filtration using reverse osmosis is one of the technologies available to combat water scarcity by increasing freshwater supplies (1). Due to advances in membrane material and operation, membranes have become more economical, and separation performance has increased (2). However, membranes are still prone to fouling, which is the loss of membrane performance due to particle accumulation on the membrane. Biological fouling (biofouling) is caused by microorganisms that form biofilms on the membrane. The microorganisms that cause biofouling have been identified, but the reasons why specific microorganisms cause biofouling are poorly understood (3).

*Sphingomonadaceae* (or sphingomonads) belong to the *Proteobacteria* and constitute a group of bacteria that are widespread in nature due to their physiological and metabolic versatility (4). *Sphingomonadaceae*, especially those belonging to the genus *Sphingomonas*, are initial membrane colonizers that remain dominant within the developing membrane biofilm (5). Here, we present the draft genome sequence of *Sphingomonas* sp. strain Sph1(2015), which was isolated from a fouled membrane using a *Sphingomonas* selective medium (6). *Sphingomonas* sp. strain Sph1(2015) is 99% similar to *Sphingomonas yabuuchiae* A1-18, *Sphingomonas parapaucimobilis* JCM 7510^T^, and *Sphingomonas sanguinis* NBRC 13937, based on 16S rRNA gene comparison.

Sph1(2015) was grown aerobically in R2A broth (Teknova, York, United Kingdom) before the genomic DNA was isolated using the UltraClean microbial DNA isolation kit (Mo Bio Laboratories, Uden, The Netherlands). A sequencing library was prepared using a 300-bp MiSeq reagent kit (version 3) and sequenced using the Illumina MiSeq platform (Illumina, San Diego, CA), yielding ca. 3,100,000 paired-end reads of 300 bp, representing 225-fold coverage of the genome. The quality of the reads was evaluated with FastQC (http://www.bioinformatics.babraham.ac.uk/projects/fastqc/). To remove the adapter sequences, Trimmomatic (version 0.36) was used to trim the raw reads to a length of 290 bp and remove the first 20 bases. Low-quality reads were removed by Trimmomatic, with a sliding window quality cutoff of Q20 (7). Trimmed reads were assembled using SPAdes (version 3.9), with k-mer sizes of 21, 33, 55, 77, 99, and 127 and the “--careful” option (to reduce mismatches and short indels) (8). Genome completeness was assessed using CheckM with *Sphingomonas* as the marker lineage, which estimated a completeness of 99.52% and a contamination of 2.78% (9). Functional annotation was performed using Prokka (version 1.12 beta) (10), with a data set for *Sphingomonas* based on 12 available genomes.

Prokka identified 3,680 protein-coding sequences, 5 genes encoding rRNA (3 5S rRNA, 1 16S rRNA gene, 1 23S rRNA), 54 tRNAs, and 1 transfer-messenger RNA (tmRNA). The total draft genome is approximately 4.1 Mbp, has a G+C content of 66.33%, and has an *N*_50_ of 71,490 bp. The final assembly of the draft genome contains 145 scaffolds. This draft genome sequence shows that Sph1(2015) carries genes for the production of Cpa, an adhesin commonly located at the pilus tip, as well as the tight adherence protein TadB/C. Sph1(2015) requires these proteins to initiate attachment to establish strong binding to the membrane surface.

### Accession number(s).

The *Sphingomonas* sp. strain Sph1(2015) genome has been deposited at DDBJ/ENA/GenBank under the accession no. MQUI00000000. The version described in this paper is version MQUI01000000.
